# Groundbreaking Anticancer Activity of Highly Diversified Oxadiazole Scaffolds

**DOI:** 10.3390/ijms21228692

**Published:** 2020-11-18

**Authors:** Alessandra Benassi, Filippo Doria, Valentina Pirota

**Affiliations:** Chemistry Department, University of Pavia, Via Taramelli 10, 27100 Pavia, Italy; alessandra.benassi01@universitadipavia.it (A.B.); filippo.doria@unipv.it (F.D.)

**Keywords:** oxadiazoles, bioactive heterocyclics, anticancer agents, telomerase, Carbonic anhydrase, histone deacetylase, kinases, tubulin, DNA, G-quadruplex

## Abstract

Nowadays, an increasing number of heterocyclic-based drugs found application in medicinal chemistry and, in particular, as anticancer agents. In this context, oxadiazoles—five-membered aromatic rings—emerged for their interesting biological properties. Modification of oxadiazole scaffolds represents a valid strategy to increase their anticancer activity, especially on 1,2,4 and 1,3,4 regioisomers. In the last years, an increasing number of oxadiazole derivatives, with remarkable cytotoxicity for several tumor lines, were identified. Structural modifications, that ensure higher cytotoxicity towards malignant cells, represent a solid starting point in the development of novel oxadiazole-based drugs. To increase the specificity of this strategy, outstanding oxadiazole scaffolds have been designed to selectively interact with biological targets, including enzymes, globular proteins, and nucleic acids, showing more promising antitumor effects. In the present work, we aim to provide a comprehensive overview of the anticancer activity of these heterocycles, describing their effect on different targets and highlighting how their structural versatility has been exploited to modulate their biological properties.

## 1. Introduction

Heterocyclic compounds are recognized as intriguing scaffolds to incorporate in bioactive small molecules, due to the crucial role that heteroatoms cover in physiological processes. Indeed, more than 85% of biologically active compounds bear at least one heterocyclic moiety [1].

In this context, oxadiazoles are small five-membered heterocycles, composed of two carbon, one oxygen, and two nitrogen atoms, which attracted a lot of interest in different scientific disciplines: from medicine [2,3] and agrochemistry [4] to materials science [5,6]. 

**Figure 1:**
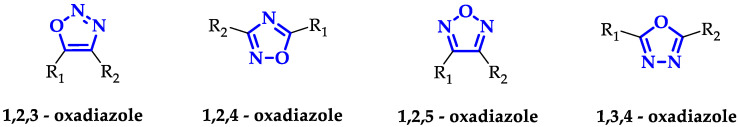
Chemical structures of the four isomers of the oxadiazole.

Their aromatic flat surface is effective in the target binding, through π-stacking interactions, or to properly outdistance the substituents according to a specific orientation [7]. Depending on nitrogen atoms position, oxadiazoles exist in four different regioisomeric forms (Figure 1): 1,2,3-, 1,2,4-, 1,2,5-, and 1,3,4-oxadiazoles.

Among the four isomers, 1,2,4- and 1,3,4-oxadiazoles frequently occur in a large series of drug-like molecules [8], including antiviral [3,9], antihypertensive [10], anti-diabetic [11], anti-inflammatory, and analgesic [12], as well as anticancer compounds [13,14]. Probably, this is a direct consequence of the orientation of the side chains (-R_1_ and -R_2_ in Figure 1), very similar for 1,2,4- and 1,3,4-oxadiazoles with respect to 1,2,3- and 1,2,5-oxadiazoles. Indeed, small bioactive molecules with resembling shapes usually show target-binding similarity that translates into the same biological effects [15]. Moreover, their bioisosteric correspondence with ester and amide groups makes oxadiazoles an interesting synthetic alternative to avoid intrinsic molecular instability (e.g., due to hydrolysis reactions), guaranteeing different hydrogen-bonding potentials with the receptors [8]. 

**Figure 2:**
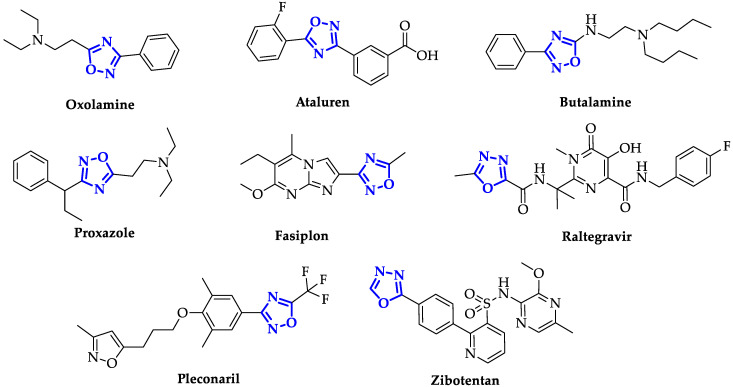
Structures of some of the most famous ligands bearing oxadiazole moiety.

During the last decades, the design of new oxadiazole-based scaffolds accelerated in medicinal chemistry, bringing most of these compounds to the preclinical stage or, even, to commercialization. Among these, the most commercially available drugs are Oxolamine, a cough suppressant [16], Ataluren, indicated for the treatment of Duchenne muscular dystrophy [17] and cystic fibrosis [18], Butalamine, a vasodilator, Proxazole, a drug for functional gastrointestinal disorders, Fasiplon, an anxiolytic drug [19], Raltegravir, an antiretroviral drug used to treat HIV [20], which has been recently proposed as repurposing drug against SARS-COV-2 [21], as well as the antiviral Pleconaril selected for the SARS-COV-2 spike protein [22] (Figure 2). Equally well known is Zibotentan, an anticancer drug in late-stage clinical trials (www.ClinicalTrials.gov Identifier: NCT00554229), which is a candidate in development by AstraZeneca (Figure 2).

Considering the meaningful social impact of cancer, due to the incessant raise incidence, with resistance towards existing drugs and severe side effects, the need to develop novel anticancer drugs is compelling.

Herein, we present a comprehensive overview of the most recent small molecules bearing oxadiazoles moieties, which act as anticancer agents. This work aims to highlight the biological targets, if known, with whom these small molecules interact to achieve their antiproliferative effects.

## 2. Enzyme Interactions

Tailor-made inhibitors for a specific enzyme result in extremely interesting as anticancer agents because of the high specificity of enzymatic activity [23,24,25]. Starting from this consideration, here we reported the results obtained in enzyme inhibition by using oxadiazoles as biological ligands.

### 2.1. Specific Inhibitors of Telomerase

Telomerase, a ribonucleoprotein enzyme, maintains the length of chromosomes catalyzing the addition of hexameric telomeric repeats (TTAGGG) to the 3′-end of the template strand of DNA. It follows that its role is necessary for the long-term viability of the cells. 

Whereas its activity is negligible in most human somatic cells, telomerase is overexpressed in around 90% of cancer cells, actively contributing to their immortality and tumor genesis [26]. Therefore, inhibition of telomerase is considered a very interesting approach for anticancer drug design.

**Figure 3:**
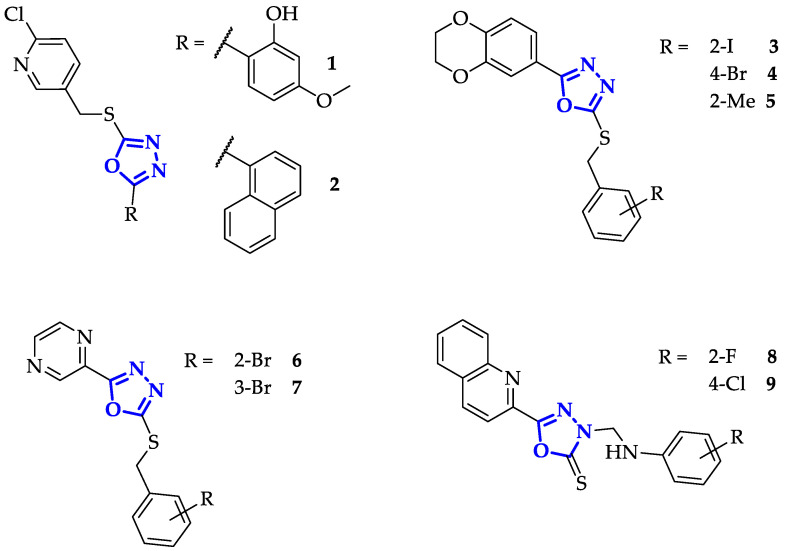
Chemical structures of 1,3,4-oxadiazole derivatives identified as good telomerase inhibitors.

In this context, Zheng et al. synthesized a family of twenty-one 2-chloropyridine derivatives bearing 1,3,4-oxadiazole units, differently substituted in position five, to evaluate their antitumor potency. Sixteen of these compounds showed a remarkable antiproliferative activity against SGC-7901 gastric cancer cells, eight with IC_50_ values comparable to the positive control (5-Fluorouracil) and eight 50% lower [27]. Among them, the best results were reached by compounds **1** and **2** (Figure 3), which exhibited the most potent activity (IC_50_ = 1.61 ± 0.06 µg/mL and IC_50_ = 2.56 ± 0.11 µg/mL, respectively). Moreover, **1** showed a specific telomerase inhibitory activity (IC_50_ = 2.30 ± 0.07 µM) identified by TRAP assay on a SGC-7901 cell extract, comparable to the positive control ethidium bromide (IC_50_ = 2.50 ± 0.23 µM) [27]. This result prompted the authors to carry out molecular docking studies (GLIDE protocol, Schrodinger Inc.) to elucidate the specific interactions between the compound and ATP binding site of the enzyme. Using the 3DU6.pdb telomerase structure deposited [28], they demonstrated the implication of three hydrogen bonds: two between the hydroxyl group of **1** and the amino group of Lys249 as well as oxygen atom of Asp344, the third between Gly391 residue and the oxygen atom of methoxyl group [27].

A very similar study was conducted by Zhang et al. on a series of 1,3,4-oxadiazole derivatives possessing a 1,4-benzodioxan moiety, previously recognized as a fundamental unit to guarantee good bioavailability and low cytotoxicity in other potential anticancer drugs [29].

All the synthesized compounds revealed a broad-spectrum antitumor activity against HepG2, HeLa, SW1116, and BGC823 cell lines according to standard MTT-based colorimetric assay, with an IC_50_ value at least 55% lower than positive control (IC_50_ = 110 µM for 5-Fluorouracil). The best antiproliferative effectiveness was obtained on HepG2 line by compounds **3** and **4** (Figure 3) with IC_50_ values of 7.21 µM and 8.54 µM, respectively. Besides, sixteen of these molecules displayed a telomerase inhibitory activity (IC_50_ concentration range of 1.27 ± 0.05–5.89 ± 0.35 µM evaluated via TRAP-PCR-ELISA assay) against SW1116 cancer cell higher than positive control Staurosporine (IC_50_ = 8.32 ± 0.08 µM). Among these, **5** showed the highest telomerase inhibition, and therefore the simulation of its binding mode on ATP binding site of telomerase (3DU6.pdb) was predicted by molecular docking studies (AutoDock software package 4.0) [29]. The oxadiazole is involved in a hydrogen bond with the amino residue of Lys372, while 1,4-benzodioxan unit gets a second hydrogen bond with hydrogen of Lys406. Moreover, three π-cation interactions shaped, two between Lys406 and 1,4-benzodioxan as well as the other benzene ring, while the third was provided from oxadiazole and Lys372 [29].

To extend their work, the same group synthesized and studied a new family of 1,3,4-oxadiazole ligands containing a pyrazine moiety [30]. Biological activity was evaluated on the same four cancer cell lines, highlighting a broad-spectrum of antiproliferative effects. In this case, the best results were obtained on HepG2 and SW1116 cells with compounds **6** and **7** (HepG2, IC_50_ = 4.22–5.79 µM; SW1116, IC_50_ = 2.46–5.06 µM; Figure 3), which are more or equally efficient than positive control Staurosporine (HepG2, IC_50_ = 6.73 µM; SW1116, IC_50_ = 4.95 µM). Besides, their telomerase inhibition against HepG2 cells was slightly higher than the positive control (IC_50_ of around 3.5 µM versus 4.14 µM), demonstrating that their antiproliferative activity was strictly related to telomerase inhibition. In particular, it has been predicted that compound **6** interacts with telomerase binding site through five hydrogen bonds: three involved oxadiazole moiety and Arg194 as well as Gln308 residues, the other two were correlated to pyrazine’s nitrogen atom and Lys189 [30]. Outcomes from molecular docking (by Discovery Studio 3.1) emphasized the positive involvement of 1,3,4-oxadiazole units in the enzyme binding and inhibition, further evidencing their specificity and potency.

A wide-ranging antitumor activity was subsequently attained by conjugating quinoline to 1,3,4-oxadiazole [31]. Among the compounds synthesized by Sun et al., seven showed in vitro anticancer activity higher than 5-fluorouracil against human tumor HepG2, SGC-7901, and MCF-7 cell lines. Distinctly, **8** and **9** (Figure 3) revealed the best IC_50_ values, with exceptional effects against HepG2 line (IC_50_ = 1.2 ± 0.2–0.8 ± 0.2 µM versus 5-fluorouracil IC_50_ = 21.9 ± 1.4 µM). Their effectiveness was closely related to telomerase inhibitory activity, as demonstrated by TRAP-PCR-ELISA assay. IC_50_ values were one order magnitude lower (IC_50_ = 0.8 and 0.9 µM for **8** and **9** respectively) than the one achieved with Staurosporine (IC_50_ = 8.3 µM) [31]. While for **8**, the interaction with the telomerase binding site is unconnected to the presence of oxadiazole unit, the 1,3,4-oxadiazole in **9** is responsible for two H-bonds that involved Gln308 and Arg194 residues, as predicted by automated docking studies [31].

### 2.2. Selective Inhibition of Carbonic Anhydrase Isoforms

Human carbonic anhydrases (CA) comprise a family of metalloenzymes that catalyze the reversible hydration of carbon dioxide to hydrogen carbonate with the release of a proton. This biological process maintains acid–base balance in the intra- and extracellular regions in several tissue and organs [32].

Among them, the membrane-bound CA IX and CA XII are overexpressed in solid and metastatic tumors, playing an important role in tumorigenesis, cancer cell signaling, and survival under hypoxic conditions and acidic extracellular environment. For these features, the selective inhibition of specific CA isoforms became a groundbreaking target in anticancer therapy.

**Figure 4:**
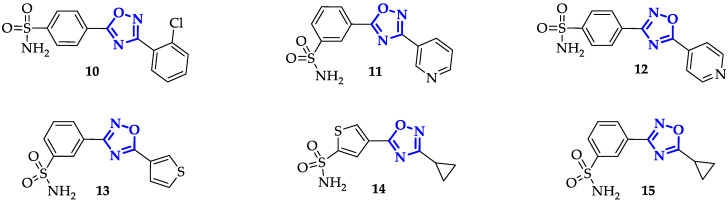
1,2,4-oxadiazoles derivatives that showed a good inhibition of carbonic anhydrase IX and XII isoforms.

Krasavin et al. synthesized, in 2018, a promising series of CA inhibitors based on primary sulfonamide-tagged 1,2,4-oxadiazole core. Through CO_2_ hydration stopped-flow biochemical assay, they compared the ligands interactions with two cytosolic CA isoforms (I and II) and two membrane-bound CA (IV and IX) [33]. Several of these compounds revealed a marked selectivity in CA II and CA IX inhibition, classical targets for treatment of glaucoma-related intraocular hypertension and solid tumors, respectively. Nineteen compounds inhibited CA II in a sub-nanomolar range, among which **10** (Figure 4) shown the best Ki value equal to 0.48 nM, twenty-five times lower than positive control acetazolamide (12.1 nM). CA IX inhibition was reached at low-nanomolar range by fourteen compounds, with the best result derived from **11** (Ki = 1.2 nM; Figure 4) significantly lower than Acetazolamide (25.8 nM) [33]. Despite the fact that these compounds did not show a univocal selectivity, the cellular localization of CA II and CA IX is so different that it was not a real disadvantage. Docking studies predicted that 1,2,4-oxadiazole unit was not involved in crucial direct interaction with the targets, except for the possible presence of H-bond with Gln92. Anyhow, it properly orients its substituents allowing them specific interactions with the targets.

Encouraged by these results, the authors developed a new family of 1,2,4-oxadiazole derivatives, containing aryl-sulfonamide units, which were effective against not only to CA II and CA IX in a sub-nanomolar range but also to CA XII in a nanomolar range [34]. By CO_2_ hydration stopped-flow biochemical assay, they compared again the inhibitory effects on CA I and II versus the two cancer-related CA isoforms IX and XII. Among the twenty-four compounds, twenty retained sub-nanomolar to low nanomolar inhibitory potency against CA II isoform, with Ki values of 0.3 nM and 0.38 nM in presence of compounds **12** and **13** respectively (Figure 4) [34], even better effects than the previous study [33]. Sub-nanomolar range was achieved on CA IX by twelve derivatives, at least one order of magnitude lower than previously reported series [33]. An exceptional result was obtained with **14** (Ki = 0.089 nM; Figure 4), around two orders of magnitude lower respect **11**. Besides, an interesting inhibition of the membrane-bound isoform CA XII, with an IC_50_ of around 10 nM was gained. In the same work, the author tested the ability of their 1,2,4-oxadiazole derivatives [33,34] to affect the cell culture viability of non-cancerous human retinal pigment epithelial cell line (ARPE-19) in comparison to pancreas ductal adenocarcinoma cell line (PANC-1). The analysis was conducted in presence of 50 µM compounds under chemical hypoxic conditions, to closely reproduce the solid tumors environment, turning out seven compounds (included **13** and **14**) more effective towards PANC-1 (30% more cytotoxic) than ARPE-19. Therefore, the most promising compounds were evaluated in normoxic and hypoxic conditions, comparing ARPE-19 with the two cancer cell lines PANC-1 and SK-MEL-2. In particular, **14** displayed selective cytotoxicity to melanoma SK-MEL-2 cells at 30 µM, while effects on normal cells were observed only at higher concentrations (100 µM). Conversely, marked cytotoxicity towards PNA-1 under hypoxic conditions was moved to **15** (Figure 4), which had shown a selective inhibition towards CA cancer-related isoform XII (versus IX) with a Ki value of 8.9 nM [34].

It is interesting to note how the replacement of 1,2,4-oxadiazol-5-yl-thiophene (**14**) with 1,2,4-oxadiazol-3-yl-thiophene (**15**) can modulate the specific selectivity versus a particular cell line.

### 2.3. Specific Interactions with Histone Deacetylase Isoforms

Human histone deacetylases (HDACs) are an enzyme family of eighteen species, which is divided into four classes. Their role is to deacetylate Lys residues on a histone, leading to more tightly wrapped DNA with less accessibility toward these transcriptionally active regions [35]. Their action is opposite to histone acetyltransferases, which transferred an acetyl group from acetyl-coenzyme A to Lys amino acids on histone proteins, increasing gene expression. Besides the histone alterations, HDACs can remove acetyl unit also from non-histone proteins, including estrogen receptors, transcription factors, and chaperons. In this sense, it interferes with several DNA processes, such as protein stability and DNA binding activity [36]. Considering that the overexpression or abnormal recruitment of HDACs are the most frequent epigenetic alterations in tumor onset and progression, inhibition of HDACs has been considered a promising strategy to fight cancer spread [37]. Here we reported some examples in which oxadiazole units participate in HDACs’ binding, inducing a class-selective and isoform-specific inhibition.

**Figure 5:**
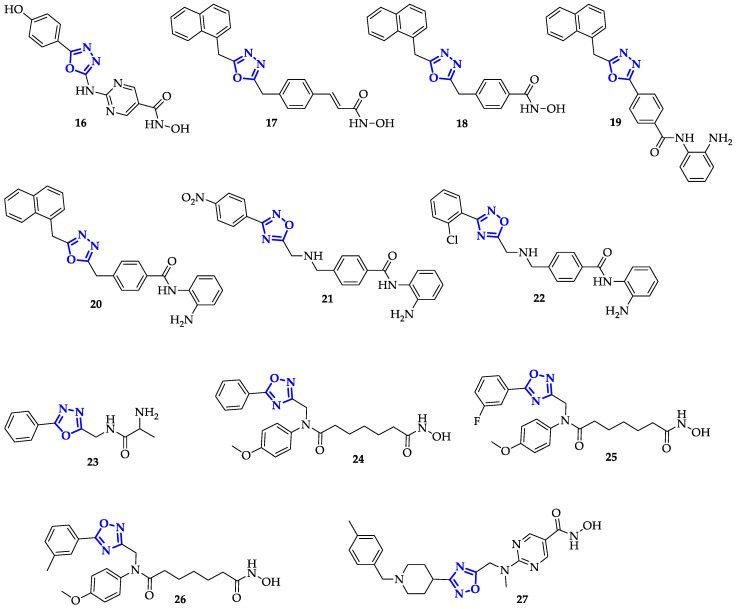
Example of oxadiazoles ligands able to efficiently inhibit histone deacetylases.

In general, the pharmacophore model of an HDAC inhibitor is constituted of three different parts: a binder of Zn^2+^ ion, a linker region to accommodate the Zn-coordinator into the catalytic binding site of HDAC and a cap group used as selective vector. Rajak et al. synthesized hydroxamic acids, containing 2,5-disubstituted 1,3,4-oxadiazole as surface recognition cap group, which resulted in a moderate anticancer activity due to specific HDAC1 inhibition [38]. In particular, **16** (Figure 5) displayed an inhibitory activity against HDAC1 (IC_50_ = 0.017 µM) at least equal to positive control Trichostatin A (IC_50_ = 0.016 µM) and an IC_50_ of 0.28 µM against human colon cancer cell (HCT-116) proliferation. Moreover, testing its activity in vivo against Ehrlich ascites carcinoma cells in Swiss albino mice, they measured 85.7% inhibition of Ascitic fluid and 77.7% of Ascitic cell, using Mitomycin-C as standard [38].

Valente et al. exploited 1,3,4-oxadiazole as polar connection linker in the design of a novel series of hydroxamates and 2-aminoanilides. Overall, these derivatives resulted very effective in inhibition of HDAC1, while only 1,3,4-oxadiazole hydroxamates compounds showed good results also against HDAC6 [39]. In particular, hydroxamates **17** and **18** (Figure 5) reached the highest inhibitory potency against HDAC1 with an IC_50_ of 0.20 ± 0.01 µM, comparable with positive control Vorinostat (IC_50_ = 0.30 ± 0.02 µM), the first FDA-approved HDAC inhibitor for T cell lymphoma progression treatment [40]. Moreover, the presence of cinnamic moiety, instead of benzoic ones, made **17** around two orders of magnitude more potent in the HDAC6 inhibition (IC_50_ = 0.030 ± 0.001 µM) than **18**, and twice as high as positive control (0.060 ± 0.003 µM). HDAC1 was successful inhibited also by the 2-aminoanilide derivatives **19** and **20** (IC_50_ of 0.30 ± 0.02 µM and 0.20 ± 0.01 µM, respectively; Figure 5), which, on the contrary, were not active against HDAC6. These compounds (5 µM) increased acetyl-histone H3 levels in human acute monocytic myeloid leukemia cells (U937), as well as acetyl-α-tubulin levels, according to their potency on HDAC inhibition. Moreover, they showed an induction of p21, regulator of p53-repression targets [41], greater than Vorinostat in the case of **18** [39]. Besides, **18** and **20** provided a block of U937 cell cycle, similarly to Vorinostat, displaying a dose-dependent apoptotic effect (evaluating pre-G1 peaks). In this sense, **17** revealed the best apoptotic activity in U937 cells: from 58% at 5 µM to 78 % at 25 µM (5 µM of Vorinostat induced 48% of apoptosis). The granulocytic differentiation in human leukemia U937 cells was also evaluated considering the expression of the surface antigen CD11c as a marker. For all tested compounds, the cytodifferentiation resulted lower than the positive control Entinostat (despite increased in a dose-dependent manner), except for **20** that induced an increase of CD11c expression comparable to control (around 50% at 5 µM after 48 h). Moreover, the antiproliferative activities of the best 1,3,4-oxadiazoles derivatives **17** and **20** were comparable, highlighting a more responsive proliferation inhibition in the micromolar range against the colorectal adenocarcinoma SW620 and on all the human acute myeloid leukemia tested cell lines (U937, HL60, HEL, KG1, and MOLM13). Compounds (2.5 μM) were tested also in combination with doxorubicin (0.2 μM) in U937 cells and **20** induced cell growth arrest in a major extent than vorinostat/doxorubicin combination [39]. Taken together, these data confirmed the ability of these 1,3,4-oxadiazoles conjugates to trigger apoptosis, cell differentiation, and cell growth arrest processes via HDAC1 selective inhibition. 

Following the same example, Cai et al. designed and synthesized hydroxamate and 2-aminobenzamide derivatives with 1,2,4-oxadiazole as polar connection linkers [42]. The efficacy of their series was proved against five human cancer cell lines: U937, HCT-116, human breast cell cancer (MDA-MB-231), and human lung cancer cells (A549 and NCI-H661). The MTT-based assay highlighted that only 2-aminobenzamide derivatives manifested a significative antiproliferative activity in comparison with Vorinostat and Entinostat controls, and the most notable results were obtained by compounds with electron-withdrawing group on the benzene unit. Among them, **21** and **22** (Figure 5) reached good antiproliferative activity over all the five cancer cells tested, with IC_50_ values in the micromolar and sub-micromolar ranges (against U937), comparable with the positive controls. Their antiproliferative activity was directly correlated to their ability in HDAC1 inhibition (IC_50_ values of 60 nM for **21** and 70 nM for **22**) higher than positive controls. They resulted only partially selective: indeed, lower activity was measured towards HDAC2 (IC_50_ values of 150 nM and 320 nM for **21** and **22**, respectively) and only negligible effects were recorded on HDAC8, slightly inhibited by hydroxamate derivate [42]. 

Considering that oxadiazole linkers seem to enhance HDAC inhibitory activity, Pidugu et al. developed a series of 2,5-disubstituted-1,3,4-oxadiazoles to selectively interact with HDAC8 [43]. In this work, the best interactions were observed with ligands bearing glycine/alanine residues able to bind Zn moiety. By in silico analysis, **23** (Figure 5) reached the best dock score with HDAC8 (−7.918 kcal/mol) showing strong and direct interactions with Tyr306, Gly151, as well as Phe152 binding site residues. Using recombinant purified HDAC8, it was verified the potency and selectivity of **23**, which inhibited HDAC8 in vitro with an IC_50_ of 98.0 ± 6.0 mM, fifteen times lower than Vorinostat (IC_50_ = 1480.0 ± 5.6 mM). Its moderate selectivity was proved by testing the activity of **23** on immunoprecipitated class I HDAC enzymes from MDA-MB-231 cell lysates. **23** (100 nM) selectively inhibited HDAC8 (53%) liken HDAC1 (30%), HDAC2 (19%), and HDAC3 (0%) [43]. Moreover, the MTT assay proved that **23** was the most active compound (IC_50_ = 230 nM) against breast cancer MDA-MB-231 cells, with an antiproliferative potential more than one order of magnitude higher than Vorinostat (IC_50_ = 6000 mM). Nevertheless, comparable sub-micromolar results were obtained from all the glycine/alanine derivatives here studied [43].

Based on the inhibition of class I HDAC, and specifically against HDAC1, Yang et al. designed a family of 1,2,4-oxadiazoles containing bi-substituted aromatic amides [44]. In general, all compounds showed good inhibition of HDAC1 (inhibition percentages were above 83% at 20 nM) compared to vorinostat (60%). Among them, three compounds—**24**, **25**, and **26** (Figure 5)—inhibited HDAC1 by 90%, showing promising activities with IC_50_ values in the nanomolar range, slightly lower than the positive control. In this context, **24** appeared to be the most active against HDAC1 with an IC_50_ of 8.9 nM, two-fold lower than Vorinostat (IC_50_ = 15 nM). Testing the antiproliferative activity of these three compounds against human hepatocellular carcinoma cells (HCCLM3 and HepG2) no significant improvements were identified for HCCLM3 cells (IC_50_ in the micromolar range), while **24** and **25** showed IC_50_ values of 1.07 µM and 1.03 µM, respectively, against HepG2 cells (fourfold lower than control) [44]. Accordingly, the authors evaluated the acetylation level of histone H3 and H4 in HepG2 cells as a response to HDAC inhibition, confirmed by a marked dose-dependent upregulation, due to ligands presence. In particular, the best results were obtained by **25** for the H3 acetylation [44].

Synthesis of four different libraries of 1,2,4-oxadiazoles, as cap group, bearing one N-substituted piperidine and connected to a hydroxamate group were proposed by Yang et al. during the same year [45]. They tested the inhibition potency towards HDAC1 and HDAC6, evidencing a major efficiency in HDAC6 downregulation (>58%) for their first compounds’ family and higher HDAC1 inhibition for the fourth compounds’ family (>62%). In this series, compound **27** (Figure 5) showed the best selective HDAC1 inhibition of 96% (inhibition of HDAC6 of 71%). Antiproliferative activity of **27** towards Raji cell line was so outstanding (IC_50_ = 12.1 ± 1.9 nM) that the compound was also evaluated towards other HDAC isoforms (1–11), arising effective against class I HDAC1, 2, and 3 with IC_50_ values in the nanomolar range (IC_50_ of 1.8, 3.6, and 3.0 nM, respectively), one order of magnitude lower than vorinostat. Therefore, in vitro antiproliferative activity of **27** was estimated towards 12 tumor cell lines, including solid and hematological tumors, and three primary AML cell lines. IC_50_ values ranged from 9.8 to 44.9 nM, a very promising result considering that the lower IC_50_ value obtained with Vorinostat was above 500 nM [45]. Acetylations of histone H3 and α-tubulin in the MM1S cells were upregulated by **27** in a dose-dependent manner, and, at low **27** concentrations, the relative expression was higher than vorinostat treatment. Moreover, **27** induced G1 cell cycle arrest in Jeko-1 cells with 73.1% of cell apoptosis after 48 h at 1 µM compound. These results highlighted that **27** is a promising 1,2,4-oxadiazole derivative, acting as a potent anticancer agent. Therefore, after checking its excellent bioavailability in SD rats, the antitumor potency of **27** was determined on a Burkitt’s lymphoma Daudi xenograft model. The compound was able to reduce tumor growth by around 50%, increasing the expression of acetylated histone H4 in tumor tissue and leading to reduced tumor proliferation and increasing DNA damage of tumor cells [45].

### 2.4. Efficient Kinases Inhibitors

Human kinases are enzymes whose role is to transfer one γ-phosphate group from ATP to a specific substrate. The phosphorylation of a biological entity can affect its activity, then, for this reason, kinases are involved in many cellular pathways essential to human physiology, including protein regulation, cell signaling and metabolism. The direct consequence is their association with the human cancer initiation and progression, making these enzymes one of the most important pharmaceutical targets in clinical cancer therapy [46]. In this context, the most recent oxadiazoles derivatives were designed and studied as inhibitors of focal adhesion kinase (FAK) [47], phosphoinositide 3-kinases (PI3Ks) [48] and different receptor tyrosine kinases [49,50,51].

**Figure 6:**
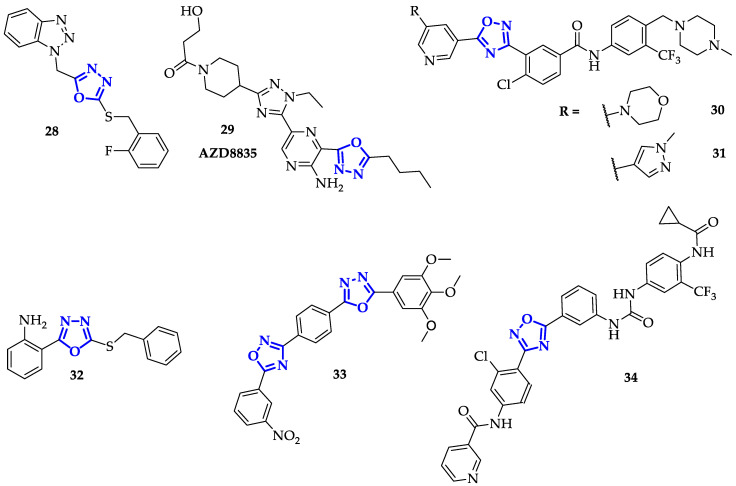
Oxadiazoles ligands that exploited kinases inhibition to reach an anticancer activity.

Zhang et al. identified an efficient FAK inhibitor, **28** (Figure 6), testing a family of compounds whose chemical structures were based on 1,3,4-oxadiazoles conjugated to benzotriazole unit [47]. They evaluated their anticancer activity against MCF-7 and HT29 cell lines using Cisplatin as a reference drug. Compound **28** showed the best antiproliferative activity against MCF-7 cells (IC_50_ = 5.68 µg/mL), twice as higher as positive control (IC_50_ = 11.20 µg/mL), and moderate activity against HT29 cells (IC_50_ = 10.21 µg/mL), slightly lower than Cisplatin (IC_50_ = 15.83 µg/mL) [47]. Despite most of the designed compounds displayed high FAK inhibitory potency, only **28** resulted to be eightfold more effective than Cisplatin (IC_50_ of 1.2 ± 0.3 µM liken 8.6 ± 0.2 µM). Moreover, its apoptotic effect on activated MCF-7 cells resulted to be dose-dependent, reaching 61.29% on MCF-7 cells with 20 µg/mL of **28** for 24 h. These promising results were in perfect accordance with the predicted interactions between **28** and the active binding site of FAK (PDB code: 2ETM). The nitrogen atom of oxadiazole moiety instituted a hydrogen bond with Cys502, while the benzene ring formed a π-cation interaction with Lys454 [47].

Another interesting kinase target is a group of lipid kinases, PI3Ks, whose biological activity resulted in protein kinase B (AKT) recruitment inducing a cascade of cell signaling. Barlaam et al. optimized kinase-specific selectivity of a potent PI3Ks inhibitor developing a family of twenty-five 1,3,4-oxadiazoles derivatives [48]. Among them, the authors revealed the outstanding compound **29**, known as **AZD8835** (Figure 6), able to inhibit cancer progression by blocking the PI3K pathway components PI3Kα and β. It reached the phase I clinical trials sponsored by AstraZeneca (www.ClinicalTrials.gov Identifier: NCT02260661) to evaluate its safety and pharmacokinetics in patients with advanced solid tumors. Compounds **29** displayed superior potency and selectivity toward PI3Kα (IC_50_ = 10 nM) with good metabolic stability. A competitive selectivity was confirmed only with BMPR1B enzyme (K_d_ value of 12 nM versus 2.3 nM for wild type PI3Kα) and PI4Kα and PI4Kβ (more than 50-fold selectivity margin versus PI3Kα) outside PI3K class I enzymes. Moreover, **29** resulted to be able to specifically interact also with mutated PI3Kα enzymes (E542K, E545K, H1047R) aside from wild type PI3Kα, with the same range of K_d_ values [48]. The potency of **29** in enzyme inhibition was common for all class I PI3K enzymes: IC_50_ values in the nanomolar range for PI3Kδ and PI3Kα (wild type and mutated), one order of magnitude higher against PI3Kγ and two order against PI3Kβ. In cell-based assay, testing the ability in AKT phosphorylation inhibition, it has been discovered that **29** affected cells sensitive to PI3Kα (IC_50_ = 0.057 µM in BT474 cells) and PI3Kδ (IC_50_ = 0.049 µM in Jeko-1 B cells). Because of its good solubility and permeability, as well as low turnover in hepatocytes, the effects of **29** on AKT phosphorylation (at Ser473 residue) were followed in nude mice bearing mutant H1047R PI3Kα SKOV-3 tumor xenografts. Significant inhibition was only seen at 25 mg/kg dose, in accordance with the lower plasma concentrations after 8 h from the administration. Anyhow, in the same model, almost complete inhibition of tumor growth (93%) was measured after chronic oral administration [48].

Taking into account that oncogenic mutations can activate RET receptor tyrosinase kinase leading to its direct involvement in different carcinomas, RET inhibition may represent a promising goal for anticancer therapy. Han et al. have developed five different compounds containing 1,2,4- and 1,3,4-oxadiazoles units to increase the RET inhibitory potency of the previously synthesized Hu1-117 (IC_50_ = 2.5 ± 0.2 nM) [49]. Despite the presence of 1,2,4-oxadiazoles led to higher RET enzymatic activities than corresponding 1,3,4 regioisomers (at least one order of magnitude higher), none showed an IC_50_ lower than Hu1-117 (in which oxadiazole unit is replaced with an alkynyl spacer). Maintaining the 1,2,4-oxadiazole moiety, the authors designed and synthesized new compounds containing different phenyl and pyridine rings. **30** and **31** (Figure 6) reached good RET inhibitory activity (IC_50_ = 2.1 ± 0.1 nM and IC_50_ = 1.80 ± 0.01 nM, respectively) more efficiently than Hu1-117 and in particular, IC_50_ value obtained for **31** resulted only twofold higher than positive control Ponatinib (IC_50_ = 0.9 ± 0.2 nM). **31** efficiently block the RET signaling pathways in BaF3/CCDC6-RET cells, indeed, at 1 µM concentration it completely abolished phosphorylation of RET and of two RET-key downstream molecules (STAT3 and ERK) [49]. Moreover, **31** inhibited cell proliferation with a similar potency for BaF3/CCDC6-RET cells and gatekeeper mutant V804 M-driven cell proliferation (IC_50_ value around 400 nM) highlighting that it could be active against both these RET-driven cancer cells. These results corresponded to the binding mode of **31** on RET active sites (RET^WT/V804M^ DFG-out homology model). Oxygen and nitrogen atoms of 1,2,4-oxadiazole are involved in H-bond interaction with Asp892, while fluorine atom with Ala807. At the same time, oxadiazole orientates the 3-substituted position leading to the formation of π-cation interaction between phenyl ring and Lys758, and a π-σ interaction between phenyl ring trifluoromethyl-substituted and Phe893 [49].

Another family of receptors tyrosine kinases is represented by Epidermal Growth Factor Receptor (EGFR), a transmembrane protein tyrosine kinase activated by ligand-induced dimerization and involved in key processes such as cell proliferation, differentiation, and migration [52]. EGFR overexpression has been associated with the spread of a broad variety of tumors, including lung, ovarian, breast, prostate, and kidney [53]. Therefore, it is not surprising that several small molecules found application as EGFR inhibitors in antitumor context. Liu et al., decided to investigate the biological activity of a library of 2-(benzylthio)-5-aryloxadiazoles, differing for the substituents introduced on aryl moiety in position 5 [50]. Cytotoxicity against MCF-7, A459 and B16-F10 has been measured and compound **32** (Figure 6), characterized by the presence of a free amino group on phenyl ring, emerged as the most toxic substrate with an IC_50_ value of 1.09 µM on MCF-7 cells. The same compound showed also a pronounced inhibitory effect on EGFR (IC_50_ = 1.51 µM), coherent with the result of the antiproliferative activity test. Docking studies confirmed favorable interactions of **32** with ATP-binding pocket of EGFR, indicating the potential of identified oxadiazole scaffold as anticancer drug. 

Polothi et al. suggested to combine two oxadiazole units (1,2,4- and 1,3,4-) to enhance their anticancer activity. They synthesized various molecules, differing for the type of substituents introduced on one phenyl ring, and tested these compounds towards MCF-7, A549, and MDA-MB-231 cells [54]. In general, IC_50_ values resulted to be particularly low (below 10 µM), especially on A459 cells. However, the most toxic compound was **33** (Figure 6), which displayed an IC_50_ value of 0.34 ± 0.025 µM on MCF-7 cells. Docking studies confirmed that **33** was potentially able to ensure strong interaction with the binding site of EGFR [54]. 

In combination with EGFR inhibitors development, very recently Dokla et al. found a new 1,2,4-oxadiazole derivative able to efficiently downregulate also hepatocyte growth factor receptor (c-Met), directly involved in tumors growth and progression [51]. The presence of urea as a linker between phenyl-oxadiazole and a second phenyl ring, followed by trifluoromethyl and cyclopropylamyde substitution, allowed the design of **34** (Figure 6), which presented a broad spectrum of antiproliferative activity with an IC_50_ lower than 1 µM. Once clarified the ability of **34** in suppressing EGRF and c-Met expression, five non-small cell lung cancer cell lines with different EGRF mutational status were used to elucidate the antitumor mechanism of **34**. Compound **34** showed consistent antiproliferative efficacies against the five tested cell lines, with IC_50_ values in the range of 0.2 to 0.6 µM. Its ability in EGFR and c-Met expression downregulation was confirmed by Western blot analysis (IC_50_ values in the range of 0.2 to 0.6 µM) occurring with a decrease in AKT phosphorylation (apart from CL68 cells). Moreover, RT-PCR demonstrated that mRNA levels of EGFR and c-Met were maintained unaltered in H1975 and A549 cells, suggesting a drug effect at protein levels [51]. Guided by the good results, the authors tested the tumor-suppressive action of **34** in athymic nude mice bearing subcutaneous H1975 xenograft tumors. Compound **34** disclosed a dose-dependent suppression of xenograft tumor growth until 60% at the end of 20-day treatment with 150 mg/Kg oral administration. No acute toxicities were associated with its administration and it has been demonstrated that tumor suppression was directly correlated with EGFR and c-Met expression [51].

### 2.5. Other Targeted Enzymes

**Figure 7:**
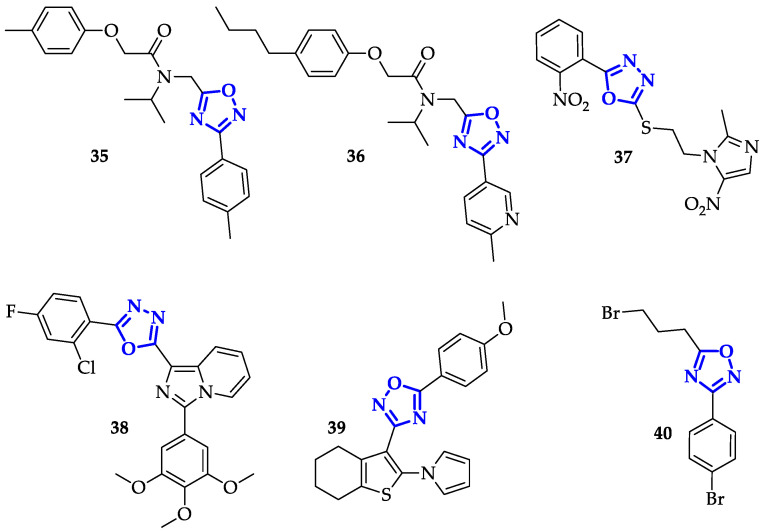
Chemical structures of oxadiazole scaffolds involved in different enzyme binding.

In addition to the previously reported studies, Ozcan et al. designed and studied a highly selective and potent inhibitor of chymotrypsin-like (CT-L) activity to impact on catalytic processes of the proteasome [55]. Starting from the identification of **35** (Figure 7) with an IC_50_ value of 0.60 µM, the authors analyzed the structure–activity relationship of novel 58 compounds reaching the most promising structure **36** (Figure 7). This 1,2,4-oxadiazole derivative showed an IC_50_ value of 0.027 ± 0.014 µM as an average of 20 determinations, 22-fold lower than **35**, with an excellent selectivity for CT-L inhibitions over both T-L and PGPH-L activities (IC_50_ > 100).

Thymidylate synthase (TS) is recognized as another attractive target in chemotherapy for its role in DNA biosynthesis. Indeed, its inhibition can lead to deoxynucleotide imbalances. The most famous antitumor agent that acts through irreversible inhibition of TS is 5-fluorouracil [56]; nonetheless, the progressive resistance to this drug of several tumors has triggered the design of new molecules. Although different successful works have been developed in this field, Du et al. proposed a series of novel 1,3,4-oxadiazole thioether derivatives, among which **37** (Figure 7) exhibited outstanding anticancer activities in vitro [57]. The authors firstly tested the anticancer activities of 1,3,4-oxadiazoles family against HepG2, SGC-7901, and MCF-7 cancer cells, highlighting the higher potency of **37** liken 5-Fluorouracil on all the cell panel, and a twofold higher IC_50_ against HepG2 (IC_50_ = 0.7 ± 0.2 µM) compared to Raltitrexed (IC_50_ = 1.3 ± 0.2 µM), both used as positive controls. Specifically, **37** appeared to be a potent inhibitor of human TS (IC_50_ = 0.62 µM) resulting 15-fold more potent than Pemetrexed (IC_50_ = 9.5 µM) and twofold less active than Raltitrexed (IC_50_ = 0.38 µM).

Another important enzyme involved in DNA biology is DNA Topoisomerase II (topo II), which controls DNA structure by catalyzing DNA cleavage and phosphodiester bonds degradation. Recently, topo II inhibitors became clinically useful as chemotherapeutical agents, and in this slice Rao et al. have developed imidazopyridinyl-1,3,4-oxadiazole conjugates, among which **38** (Figure 7) was able to affect the catalytic activity of this specific enzyme [58]. In particular, it exhibited a great growth cell inhibition in a panel of sixty cancer cell lines (mean growth of 33.54%) among which the most sensitive cell line was a non-small cell lung cancer (HOP-62). Its molar concentration required to cause 50% growth inhibition (GI_50_) were in the range of 1.30 to 5.64 µM, exhibited a broad spectrum of cytotoxicity. Subsequently, the authors verified the ability of **38** in causing cell cycle arrest at sub G1 phase till 41.70%, as well as reduction of cell population in G1 phase. Once its induced apoptotic activity was identified, increasing in intracellular ROS production was also verified, suggesting that cytotoxicity and apoptosis caused by **38** probably resulted from the mitochondrial pathway [58]. 

Conjugation of 1,2,4-oxadiazoles to thiophene, performed by Mohammed et al., led to significant toxicity on MCF-7 and HTC-116, with the identification of potent antiproliferative agent **39** (Figure 7) [59], characterized by IC_50_ value of 0.19 ± 0.05 µM on MCF-7 cells. This compound strongly inhibited topo II and, as confirmed by flow cytometry analysis, induced cell cycle arrest at G1 phase. Its proapoptotic behavior was further demonstrated by increased levels of p53 protein and other cell death modulators (Puma, Bax, and Bcl-2), measured through Western blot analysis [59]. 

Oxadiazoles showed marked biological effects also with the class Sirtuin (Sirt), a family of NAD^+^-dependent lysine deacetylases that share a conserved NAD^+^-binding and catalytic core domain [60], and are involved in critical physiological processes as regulation of transcription, genome stability and cell survival [61]. Despite the role of these enzymes is not completely clear, several Sirt-inhibitors found application as anticancer agents. A previous work of Moniot et al. evidenced specific binding interactions between Sirt2 and 3-(4-chlorophenyl)-5-(piperidin-1-ylmethyl)-1,2,4-oxadiazole, unfortunately combined with poor solubility and low bioavailability [62]. To identify novel potential bioactive compounds, they examined how modifications of the aromatic scaffold, replacement of piperidine with other amines and spacer length can affect the specificity towards Sirt2 [63]. The authors performed a preliminary screening to individuate the most potent inhibitors of the library: the percentage of residual activity of Sirt2 has been measured after the treatment with 10 and 100 µM of oxadiazoles. Compound **40** (Figure 7) was the best inhibitor, with a percentage of residual activity below 25% at 10 µM. Subsequently, the selectivity of **40** for Sirt2 has been assessed in presence of other Sirt2 isoforms, as well as was determined its ability in protein activity inhibition in presence of the substrate. Cytotoxicity of **40** was measured against a panel of cancer cell lines, including U937, NB4, HL-60, K562, and MDA-MB-231, showing a particularly high toxicity against NB4 cells at 10 µM and inducing apoptosis, above 80%, in U937 cells at 50 μM. To confirm that the observed biological activity was related to Sirt2 inhibition, NB4 and U937 cells were treated with **40** (5 and 25 µM) for 4 and 24 h, monitoring the levels of acetyl-α-tubulin. In both cases, a significant increase of acetylated tubulin has been observed, confirming the selective mechanism of action of **40** as an antitumor agent [63].

## 3. Specific Interaction with Globular Proteins: Tubulin-Binding Drugs

The constant effort dedicated to cancer research led to the identification of novel and effectiveness targets, extremely useful in the development of groundbreaking therapeutical strategies. In particular, it has been demonstrated that inhibition of several proteins could interfere with the progress of the disease. Among them, tubulin emerged as one of the most promising targets [64]. It is a filamentous protein that generates microtubules upon dimerization, which are responsible for critical cellular functions as intracellular transport, shape of cells, mitosis, polarity of cells, cell signaling, cellular integrity, and gene expression [65]. Suppression of tubulin polymerization causes an arrest of cell cycle in metaphase–anaphase transition and, subsequently, induces apoptotic cell death [66]. Therefore, tubulin inhibition has been widely exploited as a promising anticancer strategy. Several small molecules have been used for this purpose; as famous alkaloid derivatives, such as vincristine and vinblastine [67]; as well as different classes of organic compounds, including quinazolones [68], benzimidazoles [69], benzothiazoles [70], and triazoles [71]. In this context, oxadiazoles emerged as an alternative tubulin inhibitor. 

**Figure 8:**
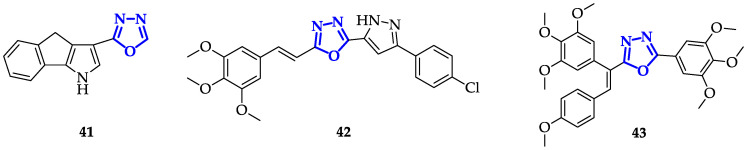
Oxadiazoles as tubulin inhibitors.

Nieddu et al. [72] synthesized different 1,3,4-oxadiazoles bearing dihydroindenopyrrole moiety in position 2, evidencing the relevance of the extension of the aromatic core for antitumor properties. They found that **41** (Figure 8) presented the highest toxicity on HeLa (IC_50_ = 0.05 µM) and MCF-7 (IC_50_ = 1.7 µM) cells, significantly increasing the percentage of cells in G2/M phase on A459, MCF-7, PC3 and SH-SY5Y cells. To assess if the observed outcomes are the results of the interaction of **41** with Tubulin, immunofluorescence analysis has been performed. Incorrect β-tubulin organization and two nuclei formation derived from 24 h incubation of **41** in HeLa cells, while inhomogeneous distribution of β-tubulin around nuclei, micronucleated cells, and decreasing in nuclei sizes were observed in MCF-7 cells. These results were in perfect accordance with the Tubulin inhibitor activity of **41** in response to its anticancer potency.

In an attempt to further improve anticancer activity of 1,3,4-oxadiazoles, Kamal et al. synthesized two series of conjugates pyrazole-oxadiazole: the first one was characterized by trimethoxy-phenyl in position 2, that was subsequently replaced, in the second series, by 3,4-(methylenedioxy)phenyl moiety [73]. As preliminary screening, the authors examined the antiproliferative activity of each derivative towards HeLa, A549, MCF-7, and IMR32 cell lines. In general, the first series of compounds showed higher cytotoxicity and, among them, **42** (Figure 8) evidenced IC_50_ values of 1.8 µM in MCF-7, 2.3 µM in HeLa, and 3.2 µM in IMR32 cells. To test its ability in inducing cycle cell arrest in G2/M phase, levels of cyclin-B1 have been analyzed after the treatment of A459 cells with 5 µM of compound for 24 h. Interestingly, **42** showed a marked increase in cyclin-B1 levels, detected through dot blot analysis. Then, the authors incubated cells with different concentrations of **42** to determine their influence on Tubulin polymerization, recording an IC_50_ value of 1.3 µM. Compound **42** significantly increased the number of cells in G2/M phase and, specifically, the treatment on A459 cells resulted in a round morphology of cells, typical of metaphase arrest, suggesting that anticancer activity of **42** could be mainly attributed to Tubulin inhibition. 

The same authors described an alternative strategy aimed to improve the anticancer activity of 1,3,4-oxadiazoles, which relies on the conjugation of these scaffolds with the double bond of Cambrestatin-A4 [74]. Different compounds were screened on HeLa, A549, MDA-MB-231 and mouse macrophage cell line B-16. Compound **43** (Figure 8), characterized by a 3,4,5-trimethoxy substituent, showed the highest efficiency with an IC_50_ value of 0.118 µM. From structure–activity relationship (SAR) analysis, it emerged that the presence of electron-donating groups on phenyl ring, conjugated to the oxadiazole, increases the toxicity. To elucidate its mechanism of action, **43** has been tested in prostate cancer cells (DU-45) to verify its effect on cell cycle. Cells were incubated with 0.5 µM **43** for 24 h and arrest at G2/M phase was observed, suggesting that **43** acts as an inhibitor of tubulin polymerization. Inhibition of polymerization has been also observed in vitro, through fluorescent analysis, evidencing 53.6% of aggregation reduction. Furthermore, incubation of DU-145 cells with **43** caused disrupted microtubule organization with spherical morphology, in direct correlation with inhibition of Tubulin polymerization. 

## 4. Nucleic Acid Structures as Oxadiazole Targets

In clinical use, many anticancer drugs directly interact with DNA. Since the 1960s, the interest in the development of this particular type of anticancer agents increased. Specifically, have been developed compounds that directly modify DNA bases, or interact through base intercalation, or, again, induce crosslinks damage. Considering their high specificity, the further discovery and characterization of such compounds is of considerable interest, therefore DNA-targeted anticancer drugs continue to be strengthened. In this context, different DNA interactive ligands bearing oxadiazole rings have been developed.

**Figure 9:**
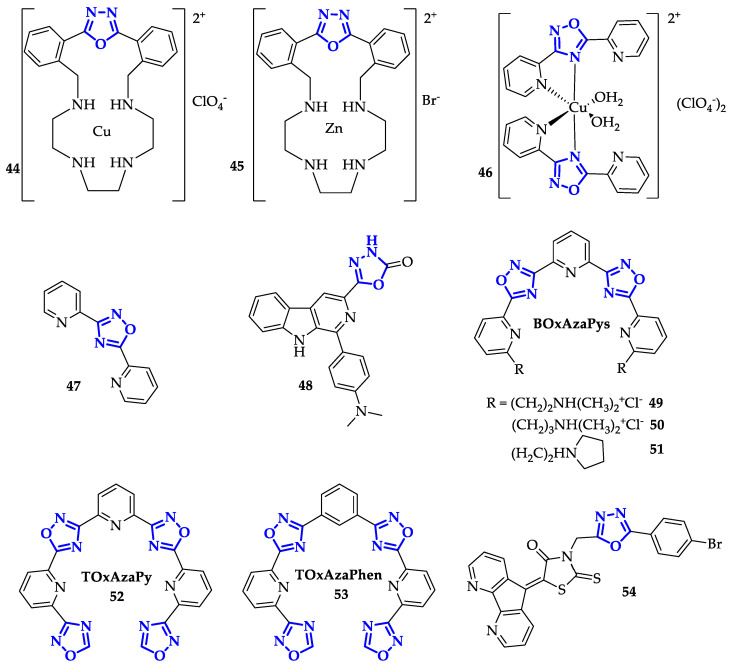
Nucleic acid ligands containing oxadiazole moieties.

Terenzi et al. taking inspiration from platinum-based chemotherapeutic compounds, synthesized two new oxadiazole metal complexes, **44** and **45** (Figure 9), able to bind DNA both by metal coordination and through intercalation of the aromatic fragments [75]. The presence of a positive charge on the metal complex enhanced the electrostatic interactions between ligands and targets, while the labile ligand with counterion can be easily substituted by a nitrogen heterocyclic atom of a DNA base forming a covalent binding. The preliminary biophysical analysis confirmed the interaction with calf thymus DNA: both zinc and copper metal complexes (**44** and **45**) determined the increase of the DNA melting temperature, the perturbation of DNA circular dichroism signal and good values of intrinsic binding constants, equal to (6.7 ± 0.5) × 10^6^ M^−1^ and (4.7 ± 0.5) × 10^5^ M^−1^ respectively. These complexes were not able to cross the cellular membrane, so biological tests were performed using a lipid carrier (Lipofectamine 2000) for internalization. The analysis led on MDA-MB231 cell line showed a good activity especially for **45** with an IC_50_ value of 100 µM. Compound **45** reduced the cell survival in a dose-dependent manner and significantly perturbed their cell cycle profile [75].

The same research group designed and developed different 1,2,4-oxadiazoles metal complexes to improve anticancer activity, exploiting an alternative DNA binding interaction. Among them, Terenzi et al. developed a new copper(II) complex with 3,5-bis(2′-pyridyl)-1,2,4-oxadiazole, compound **46** (Figure 9), to study its interaction mode with DNA and its biological activity against HepG2 and HT29 tumor cells [76]. From circular dichroism analysis and UV melting assays, **46** resulted to be comparable to typical DNA groove binders in terms of binding constant (*K*_b_ = 2.2 × 10^4^ M^−1^), DNA melting temperature (ΔT_m_ = 6.8 °C) and size of the cationic copper complex. Moreover, these spectroscopic and hydrodynamic investigations showed that while the ligand did not interact with native DNA, the copper(II) complex has the characteristics of a DNA groove binder, displaying good affinity and preserving the native B-DNA form. Biological assays demonstrated that although free ligand **47** (Figure 9) was not effective, the copper complex **46** showed similar activity on both cell lines, reducing the cellular viability of HepG2 and HT29 in a dose- and time-dependent manner (IC_50_ around 5 µM at 48 h). Moreover, cell morphology and flow cytometric analysis evidenced that the copper complex **46** caused DNA fragmentation in a significant extent amount of cells, confined in the G0/G1 phase of the cell cycle, index of apoptosis [76]. 

In contrast with metal complexes, Savariz et al. developed new entities based on β-carboline, well-known for its anticancer properties due to DNA intercalation [77], DNA binding, and DNA synthesis inhibition [78]. In a previous work, the authors improved the antitumor activity of β-carboline by introducing a thioxo-1,3,4-oxadiazolyl unit at C-3 position of carboline [79]. In an effort to achieve even better effects, they decided to replace the thioxo group with a 2-oxo derivative [80]. Antiproliferative activity was tested towards different cancer cell lines and the best outcome was achieved with **48** (Figure 9), characterized by the presence of N,N-dimethylamino group on carboline. This molecule showed particularly high toxicity on ovarian cancer cell line NCI-ADR/RES, with GI_50_ of 1.68 µM. Because of its interesting biological effect, **48** was selected to clarify its interaction with DNA, through fluorescence quenching analysis and competition assay with ethidium bromide. Potassium iodide (KI) was employed in fluorescence quenching assay to measure the occurrence of the quenching process in presence of ctDNA. Therefore, the authors measured Stern–Volmer constant (K_SV_) values for the free and the bound **48** with ctDNA (10 µM), which resulted to be 15.5 ± 1.4 L/mol and 7.15 ± 0.42 L/mol, respectively. Lower K_SV_ values recorded in presence of ctDNA compared to free molecule confirmed the ability of **48** in ctDNA interaction. Furthermore, increasing concentrations of **48** induced reduction of the emission intensity of the EB–ctDNA complex until 62% (at 20 µM **48**), evidencing that the two ligands (**48** and EB) interact with ctDNA in the same mode through intercalation [80].

B-DNA is not the only target of oxadiazole anticancer agents, other nucleic acid secondary structures, such as DNA G-Quadruplexes (G4), offer the possibility to control key biological processes in cells. In this context, Freccero’s group published, in 2012, the synthesis and the biophysical studies of a family of polyheterocyclic 1,2,4-oxadiazoles ligands (**BOxAzaPys**, Figure 9) [81]. These ligands, mimicking the well-known macrocyclic G4 binder telomestatin [82], were able to selectively interact and stabilize telomeric G4, inhibiting the activity of telomerase. This new family of acyclic G4 ligands was characterized by excellent water solubility and an unexpected potential. Indeed, **BOxAzaPys 49**, **50**, and **51** showed excellent G4 selectivity over B-DNA. Using FRET melting assay, **49**, **50**, and **51** appeared as good binders of telomeric DNA (F21T in K^+^ buffer) showing ΔTm values of 15.5, 10.5, and 16 °C, respectively, at 1 μM of ligand concentration in a large excess of B-DNA as a competitor. G4-FID and circular dichroism assays revealed that their on/off G4 binding properties were clearly controlled by their innovative structural features: the conformational flexibility and the highly hydrophobic character of the pentameric core [81]. 

These promising results prompted the same researchers to perform a structural tuning of **BOxAzaPy** ligands to enhance G4 binding. In order to achieve this goal, they decided to modulate the nature and number of the heterocycles linked to the pyridyl oxadiazole central core. Among them, more extended heptacycle-oxadiazole ligands, such as **TOxAzaPy** and **TOxAzaPhen** (**52** and **53** in Figure 9) shown high stabilization ability and a preferential binding towards the antiparallel G4 sequence 22AG (ΔTm values ranging from 13.8 to 16.8 °C, 1 μM ligand concentrations in Na^+^ buffer) with a remarkable selectivity versus a large excess of duplex DNA [83]. These new heptapyridil-oxadiazoles seem to be more effective and selective than **TOxaPy**, the benchmark heptacycle G4 ligand. The replacement of 1,3-oxazoles by the 1,2,4-oxadiazole moiety positively affected their binding affinity and led to the unexpected selectivity. Moreover, circular dichroism and G4-FID assays suggested that the introduction of 1,2,4-oxadiazole moieties induce an important change in their binding mode, indeed, they probably act as groove binders [83].

More recently Zhou et al. developed a new class of 4,5-diazafluorene derivatives bearing 1,3-disubstituted thioxothiazolidinone-oxadiazole moieties as G4 binders [84]. The lead compound **54** (Figure 9) was deeply investigated using different biophysical assays. Despite careful design, these compounds resulted to be unpromising candidates. EMSA assays suggested the formation of higher-order **54**/G4-DNA structures, but all the other experiments underlined its poor affinity (ΔTm = 4 °C, 1 μM ligand concentration, 22AG) and selectivity towards G4 structures [84].

## 5. Oxadiazoles Derivatives as Anticancer Agents without a Specific Target Identified

When a novel bioactive compound is developed, identification of its biological targets and elucidation of its mechanism of action represent one of the most challenging goals of biomedical research. This is a common issue also in the development of oxadiazole-based drugs. In this context, among the different available approaches, structure–activity relationship analysis (SAR) is the most frequent starting point, to obtain biologically active compounds. According to the studies reported in the literature, three main strategies are used to modify oxadiazole scaffolds to improve their anticancer activities: (i) conjugation to other biologically active compounds, (ii) extension of aromatic surface to enhance the binding interactions, and (iii) introduction of sulfur-based functional groups, frequently found in FDA-approved drugs [85,86].

Conjugation to already known bioactive moieties is a simple strategy to improve biological properties of a specific substrate and, on this basis, Thasneem et al. reported the synthesis of a library of oxadiazoles covalently bound to Chalcone, an unsaturated carbonyl system that showed anti-inflammatory, anti-fungal and antitumor properties [87]. However, the obtained molecules have been tested against MCF-7 cells at different concentrations (0.1, 1, 10, 50, and 100 µM) and IC_50_ ranging from 10 to 50 µM have been measured. 

Following the same approach, Li et al. synthesized a series of analogs of Imatinib, a Bcr-Abl tyrosine-kinase inhibitor employed for the treatment of chronic myelogenous leukemia [88], to individuate structural features that could further improve its anticancer activity [89]. They replaced the amide bond of Imatinib with a 1,2,3-triazole ring or 1,3,4-oxadiazole. All compounds were tested against K562, HL60 and KG-1a leukemia cell lines, however, all oxadiazole derivatives showed lower antiproliferative activity compared to Imatinib, suggesting a low antitumor potency of these structures. 

**Figure 10:**
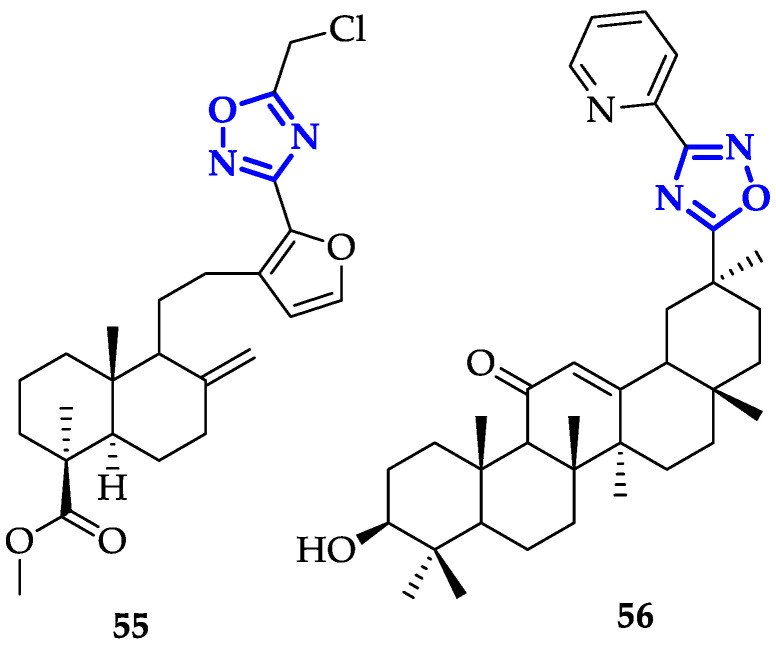
Oxadiazoles conjugated to bioactive molecules.

Modification of oxadiazole scaffolds through conjugation to bioactive natural products also represents a promising approach. Mironov et al. exploited Furano-diterpenoids of the labdane series, an anti-inflammatory and anti-allergic drug-like [90], to build novel and potent derivatives [91]. In detail, 1,2,4-oxadiazoles were conjugated to labda-8(9),13,15-triene or labda-8(17),13,15-triene core and their antiproliferative activity and GI_50_ on CEM-13, MT-4 and U937 cells were measured [91]. From this screening, compound **55** (Figure 10) emerged for its remarkable toxicity, with GI_50_ values of 0.08 ± 0.03 µM on CEM-13, and 0.35 ± 0.11 µM on MT-4. Moreover, cytofluorimetric analysis demonstrated that treatment of U937 cells with **55** induced apoptosis in 46.8% of cells after 24 h and 84.4% after 48 h [91].

Following a similar approach, Markov et al. designed a series of 1,2,4-oxadiazoles modified with 18βH-glycyrrhetinic acid (GA) [92], a pentacyclic triterpenoid with anti-inflammatory and antitumor activity [93]. In this study, the authors identified compound **56** (Figure 10) as the most toxic derivative, which differs from other molecules for the presence of pyridine bound to oxadiazole. Indeed, it showed significantly higher toxicity on Hu Tu-80 and HeLa cells, with IC_50_ of 3.8 ± 0.3 µM and 3.6 ± 0.5 µM, respectively. Compound **56** also showed marked selectivity toward cancer cell lines compared to non-malignant ones, with a selectivity index greater than 12. Investigating the ability of **56** to induce apoptosis on HeLa (Annexin V assay), the accumulation of early and late apoptotic cells (8.1% and 66.5%) was observed. Moreover, low concentrations of **56** repressed tumor cell clonogenicity and motility, while high concentrations induced cell death through caspase-dependent apoptosis. Potential antitumor activity on **56** has been examined also in vivo, in murine metastatic melanoma model: animals were treated with five injections of 50 mg/kg, and 70% reduction of metastasis growth has been observed, demonstrating that **56** represents a promising candidate in anticancer therapy.

**Figure 11:**
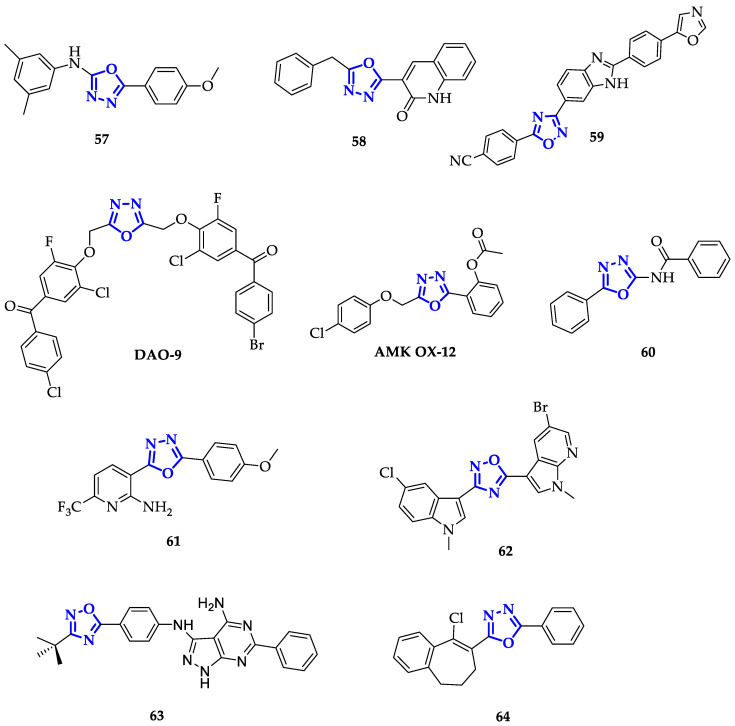
Oxadiazoles with extended aromatic system.

Despite encouraging results achieved with this approach, the modification of oxadiazole scaffolds by the conjugation of aromatic groups remains the most followed strategy. In the attempt to develop efficient anticancer agents, El-Din et al. synthesized oxadiazoles bearing a phenyl group in position 5 and a benzylic alcohol with a sulfonamide moiety in position 2 [94]. The authors evaluated the percentage of cell growth inhibition in NCl-58 cells at the concentration of 10 µM. It emerged that the introduction of p-chlorophenyl induced better efficiency (57% of inhibition) compared to other derivatives. Furthermore, the authors found that n-propylsulfonamido displayed better outcomes compared to methyl sulfonamide, suggesting that higher activity could be achieved with longer alkyl chains. Due to the interesting results obtained in this work, the same authors decided to investigate how the introduction of one or two aromatic rings in positions 2 and 5 of the oxadiazole moiety could affect the anticancer activity of this class of compounds [95]. Each molecule has been screened at a single dose of 100 µM against a panel of 60 cancer cell lines. From this screening **57** (Figure 11), emerged as the most efficient antiproliferative agent, highlighting that an extended aromatic surface (two phenyl rings with electron-donating substituents) could guarantee better anticancer activity.

Following the same approach, Salahuddin and co-workers synthesized a library of 1,3,4-oxiadiazoles conjugated to quinolines and benzimidazole derivatives and measured their growth inhibition percentage (GP) towards a broad panel of cancer cell lines [96]. Compound **58** (Figure 11) emerged for its remarkable toxicity on HTC-116, with GP equal to 7.52 µM and GI_50_ of 1.41 µM. Therefore, the introduction of quinoline moiety resulted in a promising path for the development of new anticancer drugs. 

Conjugation to benzimidazole scaffold has been also exploited by Srinivas et al., obtaining compound **59** (Figure 11), highly toxic on A549 cells, with IC_50_ of 0.12 ± 0.01 µM [97]. 

Gurupadaswamy et al. demonstrated that the antitumor activity can be improved by increasing the number of aromatic rings, despite a lower degree of conjugation. The authors identified the potent 2,5-di(4-aryloylarylox-ymethyl)-1,3,4-oxadiazole **DAO-9** (Figure 11), containing five aromatic rings, that showed high toxicity against leukemia at only 10 µM concentration [98]. To study its effect in vivo, 50 mg/kg body weight of **DAO-9** were administered in murine ascites tumor (MAC) identifying a significant reduction in tumor volume in a dose-dependent manner [99]. Furthermore, to assess its ability to induce apoptosis, MAC cells were treated with 0, 0.5, and 10 µM of **DAO-9** observing an increase in apoptosis from 10 to 21%. Moreover, treatment of MAC cells induced nuclear condensation and increased expression of p53, Bax, Bad and cyt c, a series of pro-apoptotic proteins [99]. 

Tiwari et al. synthesized a library of disubstituted 1,3,4-oxadiazoles, containing two phenyl rings with various substituents, and investigated their biological activity both in vitro and in vivo [100]. Among them, **AMK OX-12** (Figure 11) showed a significant toxic effect on Hep2 cells, with IC_50_ of 0.0007 µM after 72 h of incubation and, at the same time, displayed low toxicity (IC_50_ > 50 µM) on non-malignant cells (Chang Liver and V79). The anticancer ability of **AMK OX-12** was also confirmed by studies in vivo, in which its reduction of tumor growth in mice was comparable to Cisplatin.

Modification of the 1,3,4-oxadiazole scaffold with only two phenyl rings resulted to be a successful strategy also for Mochona et al., that individuated compound **60** (Figure 11) as the best of the series with the highest toxicity in particular on PC-3 cells (IC_50_ = 0.22 µM) [101]. Subsequently, it has been verified that **60** induced apoptosis and cell cycle arrest at G0/G1 and S phases.

Introduction of 2-substituted pyridines, as reported by Kumar et al., led to promising results as demonstrated by proving the efficiency of these compounds against HeLa, DU145, HepG2, and MDA-MB-231 cancer cell lines [102]. Among them, **61** (Figure 11) showed higher toxicity in particular on DU145 cells with an IC_50_ value of 10.7 µM. This enhanced activity has been attributed to both the presence of a trifluoromethyl group, that improves lipophilicity, and to the electron-donating effect of methoxy substituent [102]. 

On the contrary, Cascioferro et al. extended the aromatic system of 1,2,4-oxadiazole exploiting indole and azaindole derivatives with different types of substituents, including halogen atoms and methoxy units [103]. After a preliminary screening on HCT-116 cells, compound **62** (Figure 11) emerged as the most potent, displaying high toxicity on different cell lines and specifically on MCF-7 cells with IC_50_ of 0.65 ± 0.05 µM. Furthermore, authors demonstrated that **62** caused cell cycle arrest (more than 60%) at G0/G1 phase and its ability to induce apoptosis was confirmed through Annexin V/PI dual staining method, followed by cytofluorimetric analysis [103]. 

Another interesting example of modification with aza-based heterocycles was reported by Maftei et al. [104]. They synthesized different 1,2,4-oxadiazoles covalently bound to pyrimidines and evaluated their toxicity against 12 human cancer cell lines obtaining moderate to low toxicity. Best results were achieved with compound **63** (Figure 11), with IC_50_ below 9.3 µM against all tested cell lines and higher activity on ovarian adenocarcinoma OVXF 899 (IC_50_ = 2.76 µM) and colorectal carcinoma CXF HT-29 (IC_50_ = 2.90 µM) cells.

Yadagiri et al. synthesized different benzosuberones functionalized with 1,3,4-oxadiazole, 1,3,4-thiadiazole and 1,2,4-triazole moieties as alternative to commonly used aromatic rings [105]. All compounds have been tested against HeLa, MDA-MB-231, PANC1, and A549 cancer cells, and the viability of cell growth was calculated by SRB cell proliferative assay. Among them, oxadiazole derivative **64** (Figure 11) displayed the highest cell growth inhibition, with a more pronounced effect on HeLa cells (IC_50_ = 0.079 ± 0.002 µM) [105].

**Figure 12:**
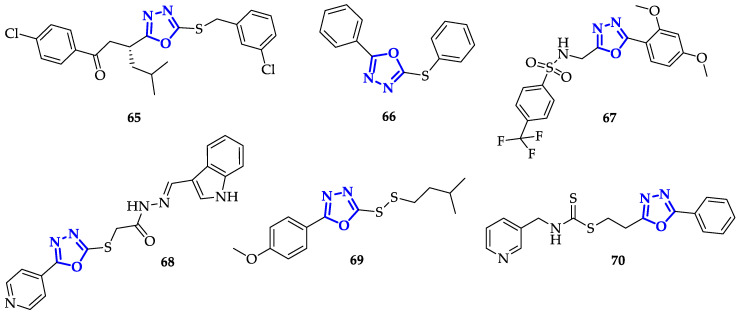
Oxadiazoles containing sulfur-based functional groups.

The presence of an aromatic system is not the only important structural feature: it has been discovered that also oxadiazoles conjugating to sulfur atoms present interesting biological properties. Guogang et al. obtained promising results after the synthesis of a library of 1,2,4-triazoles and 1,3,4-oxadiazoles, including a series of 5-thioxo-1,3,4-oxadiazole analogs, differing for the kind of phenyl substituent and the type of thio-derivative introduced on the lateral chain in position 5 [106]. Comparing 1,2,4-triazoles and 1,3,4-oxadiazole analogs, 1,2,4-triazoles have lower toxicity. Indeed, the highest antiproliferative activity was recorded by **65** (Figure 12) on K562 cells, showing an 85% inhibition ratio [106].

Yonova et al. designed a library of 1,3,4-oxadiazoles conjugated to diaryl and heteroaryl sulfides and evaluated their anticancer activity on MCF-10 and MCF-10A breast cancer cell lines [107]. The most potent compound of the series was **66** (Figure 12), with an EC_50_ value of 7.9 µM. 

In an attempt to improve both bioavailability and the antitumor effects, Kumar et al. synthesized different 2,5-disubstituted-1,3,4-oxadiazoles, containing both sulfonamide moiety and fluorine atoms, to increase lipophilicity and cellular uptake [108]. Sulfonamide groups, present in several FDA-approved drugs, were often used to improve drug-like design [86,109]. In this context, **67** (Figure 12) stood out for a drastic reduction in cell survival percentage, which was below 30% in the case of K562 cells and 42.52% and 40.68% with Colo-205 and MDA-MB 231, respectively. This result suggests that the presence of electron-donating substituents on this kind of scaffold increased anti-cancer toxicity. 

Khalil et al. synthesized thirty-six 5-pyridyl-1,3,4-oxadiazolethiols, functionalized with aliphatic, hydrazino, hydrazide, heterocyclic, or sulphonamide moieties to explore their effect against breast cancer cell lines [110]. All the tested molecules presented remarkable toxicity towards MCF-7, with IC_50_ values ranging from 0.08 to 0.01 µM. The highest efficiency was measured for compound **68** (Figure 12), characterized by an indolyl group, that evidenced, once again, the importance of an extended aromatic scaffold. 

Zhao et al. designed a library of non-symmetrical disulfides containing 1,3,4-oxadiazole scaffold and tested their antiproliferative activity against SMMC-7721, HeLa, and A549 cancer cell lines [111]. In general, only moderate toxicity was observed, except with compound **69** (Figure 12), that presented interesting selectivity towards SMMC-7721 cells, with IC_50_ = 3.40 µM. 

Another interesting functional group involving sulfur atom is represented by dithiocarbamate, recently emerged for its promising anticancer properties [112]. With the aim to improve anticancer efficacy, Li et al. synthesized novel heteroarylmethylcarbamodithioates containing 1,3,4-oxadiazole scaffold, and evaluated their biological activity on hepatoma cells Bel-7402, breast cancer cells SK-BR-3 and MDA-MB-468 [113]. During this screening, compound **70** (Figure 12) resulted to be the most toxic with marked preference for Bel-7402 (IC_50_ = 1.23 ± 0.49 µM) and SK-BR-3 (IC_50_ = 0.58 ± 0.05 µM) cells. Due to its promising activity, **70** has been further tested against other cancer cell lines, including lung cancer (H1299, H460, H522), hepatoma (Bel-7402), breast (MDA-MB-468, SK-BR-3), colorectal (COLO205, HCT-8, HCT-116, SW620) and prostate (DU145, PC3) in comparison with non-malignant cell lines (LO2 and 293). **70** presented good toxicity in all cancerous cells, with IC_50_ values from 0.39 to 7.91 µM, while IC_50_ values above 9 µM were measured in normal cells. Moreover, **70** caused chromatin condensation, a clear sign of induced apoptosis, and stopped cell cycle at G2/M phase, both in Bel-7402 and PC-3 cells. Further analysis evidenced that this substrate has a mechanism of action similar to the well-known Taxol [114]; indeed, treatment with **70** increased levels of MPM-2 and phosphorylated histone H3, two specific mitotic molecular markers that indicate mitotic arrest. This was further confirmed by the generation of Supernumerary Centrosomes and Multipolar Spindles formation upon treatment with **70**, an abnormal behavior observed also with Taxol, highlighting **70** as a promising candidate for the treatment of different kinds of tumors [113]. 

As an alternative to previously reported approaches, modification with carbohydrates is a common strategy used to improve the activity of bioactive molecules and it has been exploited also in the case of 1,2,4-oxadiazoles. Avanzo et al. reported some 1,2,4-oxadiazoles conjugated to D-ribofuranoside [115] and tested their antiproliferative activity against A549, HBL-100, HeLa, SW1573, T-47D, and WiDr cancer cell lines. However, also these compounds presented only a moderate anticancer effect, with IC_50_ ranging from 4 µM to over 100 µM.

**Figure 13:**
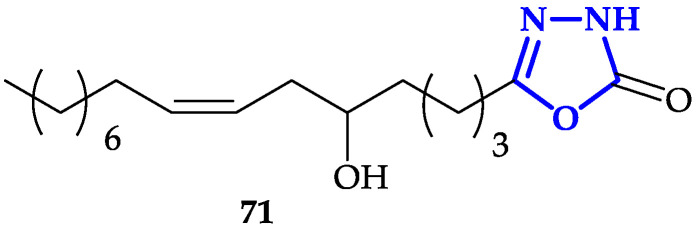
Oxadiazole conjugated to long-chain fatty acid.

Another interesting functionalization of 1,3,4-oxadiazoles scaffold is the conjugation of fatty acid analogs [116]. Hassan and co-workers described the synthesis of a library of 1,3,4-oxadiazol-2(3H)-one with fatty acids with different chain-lengths and numbers of double bonds, chosen for their promising anticancer properties [117]. Antiproliferative activity of each molecule has been tested against HeLa, MDA-MB-231, and KCL-22 (Lymphoblastoid) cells for 48 h. Authors measured IC_50_ values in the 6–50 µM range, with the highest toxicity observed in presence of **71** (IC_50_ = 6.3 ± 1.1 µM on HeLa cells; IC_50_ = 8.3 ± 1.2 µM on KCL-22; IC_50_ = 9.6 ± 1.2 µM on MDA-MB-231; Figure 13), characterized by a 16-carbon atoms chain covalently bound to 1,3,4-oxadiazol-2(3H)-one. This result underlined that the presence of a cis-double bond and a hydroxy group are two key structural features to guarantee more potent activity, despite the toxicity of **71** resulted to be lower compared to reference compound doxorubicin. Moreover, fluorescence microscopy micrographs, in agreement with previous experiments, showed that **71** was able to induce apoptosis with higher efficiency compared to other compounds of the library.

In this context, several signs of progress were accomplished in the past years as confirmed by positive results described above. However, to develop novel and specific drug-like, a lot of effort is still required to identify other modifications and structural elements that could guarantee not only high antitumor potency but also interaction with specific and defined targets.

## 6. Conclusions

The overview of oxadiazoles anticancer activity reported herein highlights the extensively use of this class of heterocyclic compounds in medicinal chemistry, further confirmed by the presence of several commercially available drugs based on these interesting scaffolds. 

The reasons for their success lie in an efficient and simple synthesis, high versatility, giving rise to elevated structural diversity, and remarkable stability, a key feature for in vivo applications. Thanks to its structural nature, oxadiazoles easily interact with bio-targets establishing π-stacking interactions or forming strong hydrogen bonds.

Despite the existence of different regioisomers, 1,2,4 and 1,3,4-oxadiazoles represent the most interesting derivatives for biological application. Recently, it has been demonstrated that oxadiazoles can interact with specific targets, as demonstrated for enzymes, globular proteins and DNA structures. This correspondence helped to clarify the origin of cytotoxicity of these compounds, allowing the improvement of drug design in relationship with the identified target. 

Furthermore, a broad number of oxadiazole ligands with promising anticancer activity have been developed despite their mechanism of action must be still clarified. It should be underlined that oxadiazoles that present relevant cytotoxicity are characterized by some common structural features: in particular, described molecules present modification with bioactive compounds, an extended aromatic surface, or the presence of a sulfur-based functional group. These results evidence the important progresses achieved in the last few years, however, a lot of effort should be dedicated to further optimize these chemical structures in order to enhance their potency and to improve the selectivity towards specific target.
